# Simultaneous Hepatic Artery and Portal Vein Thrombosis After Kasai Portoenterostomy: A Rare but Life-Threatening Complication

**DOI:** 10.7759/cureus.78855

**Published:** 2025-02-11

**Authors:** Nikolaos Ptohis, Elisavet Kanna, Eleni Batsari, Zoi Lamprinou, Ioannis Skondras

**Affiliations:** 1 Interventional Radiology Department, General Hospital of Athens "G. Gennimatas", Athens, GRC; 2 2nd Pediatric Surgery Department, Athens Children's Hospital P&A Kyriakou, Athens, GRC; 3 Nursing Department, National and Kapodistrian University of Athens, Athens, GRC

**Keywords:** biliary atresia, hepatic artery thrombosis, kasai procedure, liver transplantation, pediatric surgery, portal vein thrombosis, vascular complications

## Abstract

Biliary atresia (BA) is a progressive neonatal bile duct disease treated with the Kasai procedure; however, complications may necessitate liver transplantation. We report a rare case of simultaneous hepatic artery and portal vein thrombosis in a term infant after Kasai surgery, leading to acute liver failure and urgent transplantation. Rapid deterioration with metabolic acidosis, hypovolemic shock, and coagulopathy suggested “liver infarction,” confirmed by imaging, while thrombolysis failed to restore full viability. This case highlights the importance of recognizing vascular complications in BA and considering prophylactic anticoagulation to reduce risks. Three years posttransplantation, the patient remains stable.

## Introduction

Biliary atresia (BA) is a progressive, inflammatory, and fibrosclerotic disease of the bile ducts that occurs in the neonatal period [1,2]. The only effective treatment for BA is surgical intervention, which includes hepatic portoenterostomy (Kasai procedure) and, in some cases, liver transplantation-both of which have significantly improved survival rates in affected children [3,4]. When performed before two months of age, the Kasai procedure can restore biliary drainage in up to 90% of patients [5,6]. However, despite early surgical intervention, many patients develop progressive hepatic cirrhosis and portal hypertension, ultimately requiring liver transplantation as the only viable treatment option [3,5].

While the Kasai procedure can be effective, it is associated with a range of complications, which can be categorized as minor and major [1-3]. Minor complications include prolonged jaundice, cholangitis, and bile leaks, while major complications include portal hypertension, hepatopulmonary syndrome, and liver failure [3-5]. Among the rare but life-threatening complications are vascular complications, such as hepatic artery thrombosis (HAT) and portal vein thrombosis (PVT), which can lead to acute liver failure and necessitate urgent transplantation.

This report aims to highlight the rare occurrence of simultaneous HAT and PVT following Kasai portoenterostomy and its critical implications for clinical management. While PVT post-Kasai has been previously documented, the concurrent thrombosis of both the hepatic artery and portal vein, leading to acute liver failure and urgent transplantation, represents an exceptionally rare complication. To our knowledge, this specific presentation has not been previously reported.

Given the severity of vascular thrombosis in this case, a variety of thrombolytic strategies were considered, with an emphasis on tissue-type plasminogen activator (tPA) therapy, which was crucial for the patient’s survival. Due to the risk of hemorrhagic complications, systemic thrombolysis was avoided, and a targeted approach was utilized, successfully restoring portal vein flow, preventing further ischemic damage, and serving as a bridge to life-saving liver transplantation.

This case underscores the importance of early recognition of vascular complications following Kasai portoenterostomy, the need for individualized thrombolysis approaches, and the critical role of timely intervention in optimizing patient outcomes.

## Case presentation

A female infant, born at 38+1 weeks gestation, was admitted to the neonatal intensive care unit (ICU) for eight days due to respiratory distress. During the hospitalization, elevated levels of bilirubin (total: 6.7 mg/dL; direct: 0.5 mg/dL) were noted, as seen in Table 1. On day six of life, the infant reached a maximum bilirubin level of 16 mg/dL and was treated with phototherapy. She was discharged with instructions to undergo repeat bilirubin testing on day 22 of life.

**Table 1:** Detailed chronological summary of laboratory findings: trends in biochemical and coagulation parameters with reference ranges throughout the clinical course SGOT (AST): serum glutamic oxaloacetic transaminase (aspartate aminotransferase); SGPT (ALT): serum glutamic pyruvic transaminase (alanine aminotransferase); ALP: alkaline phosphatase; γGT: gamma-glutamyl transferase; INR: international normalized ratio; PT: prothrombin time; APTT: activated partial thromboplastin time; ICU: intensive care unit

Laboratory parameter	Value	Unit	Reference range
Initial hospitalization			
Total bilirubin	6.7	mg/dL	0.1-1.2
Direct bilirubin	0.5	mg/dL	0-0.4
Day 6 follow-up			
Total bilirubin (peak)	16	mg/dL	0.1-1.2
Day 22 follow-up			
Total bilirubin	5.8	mg/dL	0.1-1.2
Direct bilirubin	2.3	mg/dL	0-0.4
SGOT (AST)	51	IU/L	10-40
SGPT (ALT)	37	IU/L	7-56
ALP	394	IU/L	44-147
γGT	589	IU/L	7-32
Day 32 admission			
Total bilirubin	4.08	mg/dL	0.1-1.2
Direct bilirubin	3.76	mg/dL	0-0.4
Day 33 admission			
Total bilirubin	4.2	mg/dL	0.1-1.2
Direct bilirubin	3.9	mg/dL	0-0.4
Post-Kasai surgery (ICU)			
pH	7.02	-	7.35-7.45
Base deficit	-16	mEq/L	-2 to +2
SGOT (AST)	1,364	IU/L	10-40
SGPT (ALT)	382	IU/L	7-56
γGT	302	IU/L	7-32
ALP	553	IU/L	44-147
INR	1.52	-	0.8-1.2
PT	16.9	seconds	9-12
APTT	57.5	seconds	25-35
1st postoperative day			
SGOT (AST)	6,000	IU/L	10-40
SGPT (ALT)	1,300	IU/L	7-56
INR	3.78	-	0.8-1.2
Postthrombolysis			
SGOT (AST)	1,869	IU/L	10-40
SGPT (ALT)	770	IU/L	7-56
Total bilirubin	13.6	mg/dL	0.1-1.2
Direct bilirubin	7.3	mg/dL	0-0.4
INR	4.7	-	0.8-1.2

At the follow-up, laboratory testing revealed a persistently elevated direct bilirubin fraction (total bilirubin: 5.8 mg/dL, direct bilirubin: 2.3 mg/dL, serum glutamic-oxaloacetic transaminase (SGOT): 51 IU/L, serum glutamic pyruvic transaminase (SGPT): 37 IU/L, alkaline phosphatase (ALP): 394 IU/L, and gamma-glutamyl transferase (γGT): 589 IU/L).

On day 32 of life, the patient was admitted to another hospital for evaluation of direct hyperbilirubinemia and stool discoloration. Laboratory tests showed a total bilirubin level of 4.08 mg/dL and a direct bilirubin level of 3.76 mg/dL. An initial abdominal ultrasound failed to visualize the gallbladder, even after a 12-hour fasting period, raising suspicion of BA.

The patient was referred to our clinic the following day for further evaluation. On day 33 of life, laboratory results indicated persistently elevated bilirubin levels (total: 4.2 mg/dL; direct: 3.9 mg/dL) (Table 1). A repeat abdominal ultrasound performed by our hospital’s radiology team strongly suggested BA. Key findings included an absent gallbladder despite fasting, a hyperechoic fibrotic remnant (triangular cord sign) anterior to the portal vein bifurcation, and no visible common bile duct. Additionally, the hepatic artery was enlarged, suggesting compensatory arterial hypertrophy, and the liver parenchyma appeared mildly echogenic, indicative of early fibrosis. These findings, combined with persistently elevated direct bilirubin levels and acholic stools, strongly supported the diagnosis of BA and warranted further confirmation.

**Figure 1:**
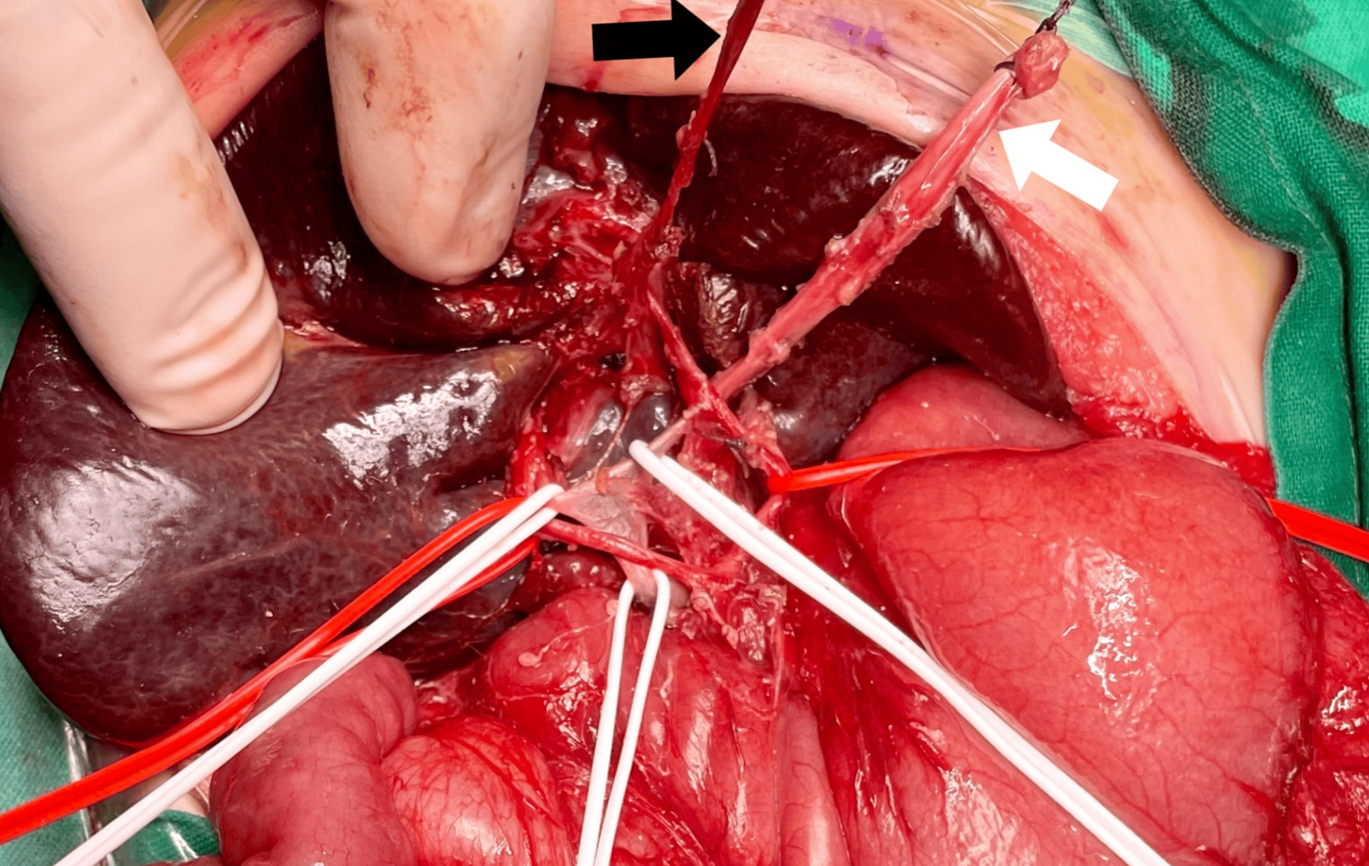
Intraoperative view demonstrating vascular slings in the porta hepatis White slings encircle the right and left portal veins, while red slings surround the right and left hepatic arteries. The white arrow indicates the round ligament of the liver, aiding in the identification of key structures, while the black arrow points to an atrophic, irregularly shaped, and hypoplastic gallbladder, a characteristic finding in biliary atresia

The patient was taken to the operating room for a typical Roux-en-Y hepatic portoenterostomy (Kasai procedure). A Chevron incision was made, but the liver was not mobilized. An atrophic gallbladder and a fibrous atrophic bile duct were noted. Vascular slings were used to embrace the right and left portal veins and the right and left hepatic arteries (Figure 1). A Roux-en-Y hepaticojejunostomy was constructed, 20 cm distal to the ligament of Treitz, using a 70-cm jejunal loop.

Upon admission to the ICU, the patient exhibited hemodynamic instability, characterized by a capillary refill time of four seconds, cold extremities, nonpalpable peripheral pulsations, and decreased urine output. Intraoperatively, the patient remained hemodynamically stable, with no significant fluctuations in blood pressure or heart rate. Temperature was actively maintained within the normal range, and no episodes of intraoperative hypothermia were recorded. However, a gradual decline in perfusion parameters was noted postoperatively, leading to progressive circulatory instability upon ICU admission. Laboratory findings showed severe metabolic acidosis (pH: 7.02; base deficit: -16), elevated liver enzymes (SGOT: 1,364 IU/L, SGPT: 382 IU/L, γGT: 302 IU/L, and ALP: 553 IU/L), and coagulation abnormalities (international normalized ratio (INR): 1.52, prothrombin time (PT): 16.9 seconds, and activated partial thromboplastin time (APTT): 57.5 seconds).

These symptoms were likely due to postoperative hypovolemia and a systemic inflammatory response, exacerbated by vascular compromise from HAT and PVT. Severe metabolic acidosis indicated poor tissue perfusion and anaerobic metabolism, while coagulopathy (elevated INR, PT, and APTT) reflected worsening hepatic dysfunction. The decreased urine output suggested renal hypoperfusion, and cold extremities with delayed capillary refill pointed to peripheral vasoconstriction secondary to shock. The combination of progressive metabolic acidosis, liver enzyme elevation, and hemodynamic instability strongly suggested evolving liver ischemia and impending acute liver failure.

By the first postoperative day, the patient’s condition deteriorated significantly, developing severe oliguria and requiring inotropic support. Laboratory tests revealed a sharp rise in liver enzymes (SGOT: 6,000 IU/L, SGPT: 1,300 IU/L) and worsening coagulopathy (INR: 3.78).

Ultrasound imaging showed disrupted liver parenchymal architecture. The main intrahepatic branches and portal vein bifurcation were dilated but exhibited no detectable flow, while low-velocity arterial flow was noted in what appeared to be hepatic artery branches. The hepatic veins also showed reduced flow velocities, and the extrahepatic portion of the hepatic artery demonstrated high contractile velocities. Computed tomography angiography confirmed thrombosis in both the hepatic artery and portal vein.

**Figure 2:**
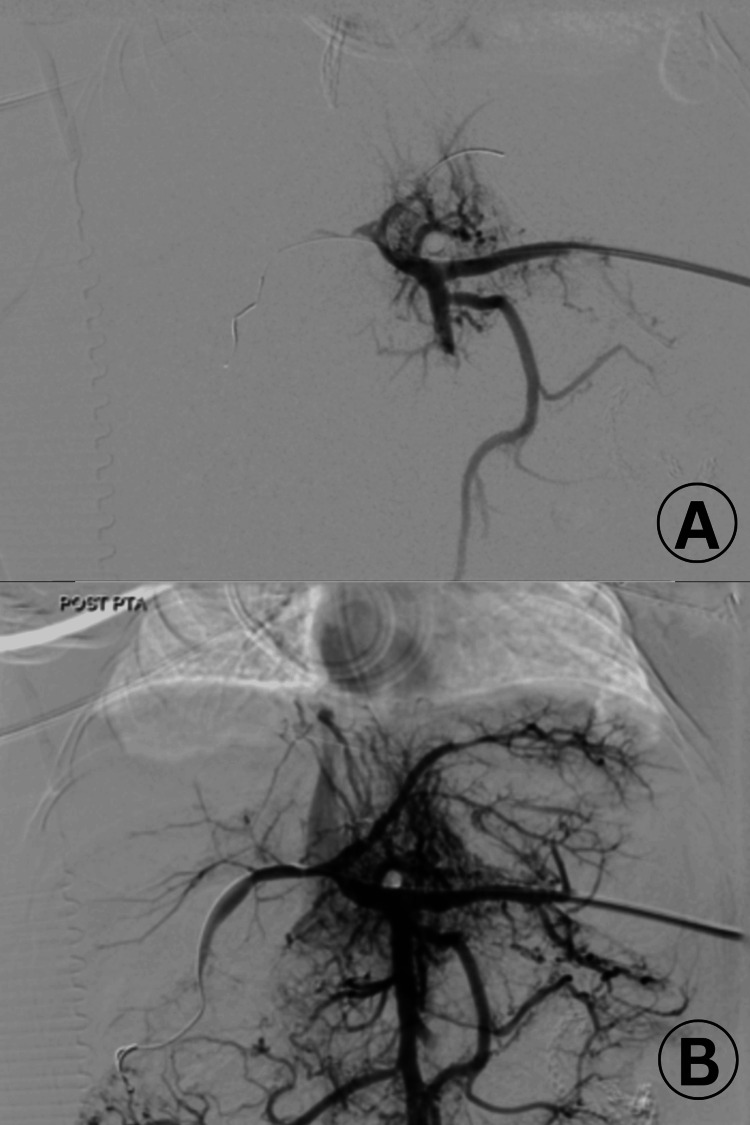
(A) Digital subtraction angiography (DSA) of the liver reveals complete occlusion of both portal vein and hepatic artery. (B) DSA performed after percutaneous transluminal angioplasty (PTA) using a 3 mm balloon and a kissing balloon technique with 1.5 mm balloons shows successful recanalization of the right and left main portal vein (PV) branches following the administration of 3 mL of recombinant tissue-type plasminogen activator (r-tPA)

The patient was transferred to the interventional radiology department, where portal vein aspiration and angioplasty were performed via a splenic vein entry point, despite an elevated INR of 4. Angiography confirmed HAT and PVT at the portal of the liver before bifurcation into the main branches. Thrombolysis in the hepatic artery was contraindicated due to a high risk of complications, as restoration of portal vein flow was prioritized to facilitate liver transplantation, a life-saving intervention. Thrombus aspiration and thrombolysis with tPA (0.5 mg/kg) successfully restored portal vein flow to the two main hepatic branches, as confirmed by follow-up ultrasonography (Figure 2).

Following thrombolysis, the patient exhibited a decrease in transaminases (SGOT: 1,869 IU/L, SGPT: 770 IU/L) but an increase in bilirubin (total bilirubin: 13.6 mg/dL, direct bilirubin: 7.3 mg/dL) and INR (4.7). Given the patient’s critical condition, the hepatologist recommended transfer to Bambino Gesu Hospital in Rome, where the patient subsequently underwent a liver transplant. The patient survived and remains in good condition three years later.

A key limitation of this report is the lack of access to the histological examination of the explanted liver and hepatic artery, as the transplant procedure was performed at a different center in Palermo. However, this case highlights the importance of early recognition of vascular complications post-Kasai and the need for prompt intervention to prevent irreversible liver damage.

## Discussion

Following a successful Kasai procedure, our patient developed metabolic acidosis and hypovolemic shock with severe deterioration observed on the second postoperative day. Imaging confirmed thrombosis of both the hepatic artery and portal vein, necessitating urgent liver transplantation as the liver was no longer viable. This rare presentation of simultaneous thrombosis in two major vessels raises important questions about its etiology, particularly given the rapid clinical decline observed. To our knowledge, this is the first documented case in the literature of total vascular thrombosis in a neonate with BA, though there are previous reports of acute liver failure in BA patients experiencing hypotension and/or hypovolemia.

PVT is uncommon in neonatal BA. Literature suggests that 40% of cases are associated with intra-abdominal infections, particularly ascending cholangitis, which may trigger intrahepatic PVT. In patients with Gram-negative bacterial infections, sepsis-induced coagulation cascade activation significantly increases the risk of PVT. Given that post-Kasai cholangitis is common, with incidence rates between 50% and 90%, this may represent a critical risk factor. Additional causes of PVT include vascular anomalies such as intestinal malrotation, situs inversus, portal vein anomalies, or absence of the inferior vena cava [1]. However, these conditions were not evident in our patient.

Traumatic injury to the portal vein during the Kasai procedure may also be considered, though such injuries are rare. Notably, other potential risk factors like thrombocytosis and polycythemia were excluded in this case [1].

Phototherapy has been identified as a common risk factor for PVT in preterm neonates [7,8]. While our patient was full-term, she underwent phototherapy due to elevated bilirubin levels, suggesting a potential association that warrants further investigation.

HAT, while a well-documented complication following liver transplantation, has not previously been reported in BA patients post-Kasai procedure [4,6,9]. Although vascular slings are routinely used during the Kasai procedure, their role in contributing to thrombosis remains uncertain and requires further exploration. The rarity of simultaneous HAT and PVT underscores the need for heightened awareness of this potential complication, as early recognition and intervention are critical to preventing acute liver failure.

## Conclusions

Kasai portoenterostomy remains the primary treatment for children diagnosed with BA. However, the sudden thrombosis of two major vessels-the hepatic artery and the portal vein-is a rare, unexpected, and life-threatening complication that warrants further investigation. Clinicians should be vigilant for the possibility of “liver infarction” when encountering signs such as increasing jaundice, elevated transaminases, and rapid liver function deterioration in a BA patient with a history of hypovolemic episodes. Prompt recognition and management are critical to preventing irreversible liver damage.

From a research perspective, vascular pathology in BA patients deserves closer examination. Although rare, thrombosis as a postoperative complication should not be underestimated. Investigating its underlying mechanisms may reveal actionable insights. Furthermore, the potential role of prophylactic anticoagulation as a therapeutic strategy for neonates with BA merits exploration. Identifying its safety and efficacy could pave the way for improved outcomes in this vulnerable population.
